# Editorial: Multimodal management of advanced gastric cancer

**DOI:** 10.3389/fonc.2022.1113856

**Published:** 2022-12-29

**Authors:** Pankaj Kumar Garg, Andreas Brandl, Luigi Marano

**Affiliations:** ^1^ Department of Surgical Oncology, Shri Guru Ram Rai Institute of Medical and Health Sciences, Dehradun, India; ^2^ Department of Surgery, Charité - Universitätsmedizin Berlin, Berlin, Germany; ^3^ Department of Medicine, Surgery, and Neurosciences, University of Siena, Siena, Italy

**Keywords:** gastric cancer, gastrectomy, neoadjuvant chemotherapy, peritoneal metastases, HIPEC (heated intraperitoneal chemotherapy), adenocarcinoma

As per the GLOBOCAN 2020 data, Gastric cancer is the fifth most common cancer and is the fourth major cause of cancer related death worldwide (1). The sheer magnitude of the gastric cancer cases has compelled the researchers across the globe to perform extensive research to demystify its etiopathogenesis to management strategies. As editors of the Research Topic on “*multimodality management of gastric cancer*”, we enjoyed this academic journey of assessing a wide variety of submitted research articles.

The incidence and treatment of gastric cancer significantly varies across the globe. East-west divide is perhaps most apparent in the management of gastric cancer. While west has uniformly adopted the neoadjuvant chemotherapy (NACT) for local advanced gastric cancer (LAGC), many centres in the east still practice upfront surgery in all operable non-metastatic LAGC followed by adjuvant chemotherapy. Which multimodal treatment is best? Based on a Propensity Score-Matched analysis of 902 patients, Xu et al. concluded that NACT leads to improved overall survival and disease free survival without compromising the postoperative outcomes. In a large retrospective analysis of 372 patients treated between 1994 to 2021 in tertiary care centre in India, Kumar et al. highlighted that multimodality treatment is warranted in LAGC. Though there was an expected heterogeneity in the neoadjuvant/adjuvant strategies, the authors reported a 3-year disease-free survival and overall survival of 36.2% and 67.8% - the 5-year disease-free survival and overall survival were 30.1% and 37.7% after a follow-up of 50.16 months. The extent of surgery has been controversial in the locally advanced gastric cancer (2), especially in those patients who achieve good response following NACT. Xu et al. advocated that addition of para-aortic lymphadenectomy to standard gastrectomy improves survival in patients who had pre-NACT para-aortic metastasis. Moreover, tumour histology largely affects surgical approach concerning the extent of lymphadenectomy. Recent studies comparing D2 with D3 dissection in a clinical setting including both prophylactic and curative super-extended dissections, showed that D3 offers a better locoregional control in advanced LAGC with diffuse histotype compared to D2 (10.1016/j.ejso.2015.01.023). However, D3 lymphadenectomy should only be performed in dedicated high-volume hospitals, due to the high risk of post-operative complications and mortality (10.1016/j.ejso.2010.03.008).

Recent improvements in surgical techniques – and locoregional control of peritoneal metastasis (PM) including hyperthermic intraperitoneal chemotherapy (HIPEC) and pressurized intra-peritoneal chemotherapy (PIPAC), as well as systemic chemotherapy has led to a paradigm change in the management of gastric cancer with PM (3, 4). Prabhu et al. presented a lucid and comprehensive review of various currently available intra-peritoneal treatment options; it generates a new hope for those patients with gastric cancer with PM, who once used to have dismal long-term survival. As the systemic chemotherapy continues to evolve, the benefit of adding surgery in a subgroup of metastatic gastric cancer (MGC) with isolated PM is being widely debated. In a retrospective series of 118 patients with MGC, who had isolated PM without any distant metastasis treated in a single centre in Italy, Morgagni et. al. reported impressive survival data in patients who had surgery plus HIPEC following systemic chemotherapy; the median OS was 60.4 months (9.2–60.4) in patients who had a complete response compared to a median OS 31.2 (15.8–64.0) who had a partial response.

Hepatoid adenocarcinoma of the stomach (HAS) is a rare type of gastric cancer that secretes alpha fetoprotein (AFP). Being a rare tumor, the management guidelines for HAS are not uniform. Zhou et al. analyzed 100 patients of HAS and concluded that upfront surgery followed by adjuvant chemotherapy seems an appropriate management strategy.

In order to further push the boundaries to improve survival in MGC with synchronous unresectable liver metastasis, Wang et al. submitted their protocol for a RCT to determine if addition of D2 gastrectomy plus radiofrequency ablation of liver lesions to systemic chemotherapy provide benefits to these patients compared to chemotherapy alone. The researchers have planned a multicentric trial with a large sample size of 200 patients. To open up another treatment frontier for gastric cancer, Zhang et al. evaluated the clinical outcomes of Human Adenovirus Type 5 (H101) combined with chemotherapy for advanced gastric carcinoma (GC) patients. Though the sample size was small, the authors highlighted that addition of H101 to chemotherapy leads to a significantly better disease control rate and overall rates compared to chemotherapy or H101 therapy alone. It definitely calls for further trials to assess if oncolytic virus therapy has a significant therapeutic potential in the management of gastric cancer.

Disease relapse – both loco-regional and systemic - is a major concern in the minds of both oncologists and patients prior to instituting the treatment. A number of clinic-pathological factors and disease stage helps us determine the chances of treatment failures. Li et al. performed a meta-analysis to evaluate the relationship between circulating tumor cells (CTC) and the prognosis of patients with gastric cancer. The meta-analysis included 14 retrospective cohort studies with 1053 patients and concluded that positive CTC suggest a poorer prognosis compared to those with CTC-negative tumors (HR=2.12, 95%CI 1.37, 3.29, P=0.0007).

A number of biomarkers are being studied all over the world for prognostication in gastric cancer. Insulin-like growth-factor-binding proteins (IGFBPs) play important roles in tumor occurrence and development by prolonging the half-life of the IGFs, controlling their access to IGF receptors (IGFRs), and promoting or inhibiting IGF downstream signaling pathways. There are two types of IGFBPs based on their different affinities for IGFs: high-affinity binding proteins (IGFBP1–6) and low-affinity binding proteins (IGFBP7–10). In a bioinformatic analysis study, Liu et al. described the involvement of IGFBPs, especially IGFBP7, in gastric cancer development through the extracellular matrix.

Financial toxicity of the treatment is another road-block in the optimum management of cancers including gastric cancer. Any oncological intervention must either lead to improvement in longevity or quality of life or both. Moreover, it must be cost effective as well so that the financial burden of the treatment for unknown/minimal benefit does not lead to bankruptcy of the caregivers destroying their social, emotional, family, and personal lives. Shu et al. evaluated the cost-effectiveness of nivolumab plus chemotherapy vs. chemotherapy as first-line therapy in patients with advanced gastric cancer/gastroesophageal junction cancer/esophageal adenocarcinoma from the perspective of the Chinese healthcare system. Their economic evaluation showed that the incremental effectiveness and cost of nivolumab plus chemotherapy versus chemotherapy alone were 0.28 QALYs and $78,626.53, resulting in an incremental cost-effectiveness ratio of $278,658.71/QALY which was much higher than the willingness-to-pay (WTP) threshold of China ($31,498.70/QALY). The authors highlighted that Nivolumab plus chemotherapy was clearly not a cost-effective treatment strategy compared with chemotherapy as first-line therapy for patients with advanced gastric cancer/gastroesophageal junction cancer/esophageal adenocarcinoma in China at current prices.

Though Trastuzumab for Gastric Cancer Trial established the role of Trastuzumab for Her2 positive gastric cancer, the benefit was marginal - 2.7 months in the overall median survival (HR 0.74, 95% CI 0.60–0.91; p value.0046) (5). In a study of 31 HER2-positive AGC patients who were treated with trastuzumab plus chemotherapy as first-line therapy for recurrent (n=8) or metastatic (n=23) tumors, Kim et al. correlated tumor mutation burden (TMB) with tumor response. The authors found that high TMB was associated with a favourable response rate compared to low TMB (75.0%, n=3/4 versus 59.3%, n=16/27).

We believe that the current research addressing all aspects of gastric cancer seems promising and provides hope for thousands of patients who are diagnosed with gastric cancer worldwide.

## Author contributions

PG wrote the initial manuscript draft. AB and LM gave critical inputs. All the authors read the final manuscript and approved it for publication.
